# The worldwide coronavirus disease 2019 outbreak: Advice and recommendation on radiology management and infection control from makeshift hospitals in Wuhan

**DOI:** 10.1097/MD.0000000000025117

**Published:** 2021-05-14

**Authors:** Lin Mu, Caijuan Zhang, Yun Pei, Jingyu Wang

**Affiliations:** a Department of Radiology, The First Hospital of Jilin University, 71 Xinmin Street; b College of Electronic Science and Engineering, Jilin University, Changchun, Jilin Province, PR China.

**Keywords:** coronavirus disease 2019, emergency shelter, infection control, tomography, X-ray computed

## Abstract

To describe and advise on management protocols and infection-protection experience of the radiology department in makeshift hospitals in Wuhan during the coronavirus disease 2019 (COVID-19) outbreak.

Based on the literature review and the experience in the frontline, we retrospectively reviewed the configuration of the radiology department, human resource, personal protection, examination procedures for patients confirmed with COVID-19 in Wuhan fangcang shelter hospital.

From February 11, 2020 to March 10, 2020, 2730 and 510 CT examinations were performed in the Hanjiang shelter hospital and Hanyang Sports School shelter hospital, respectively, including initial examinations and re-examinations. The maximum number of daily CT examinations reached 289. The CT scanned a patient approximately once every 13 mins.

Fangcang shelter radiology department could be powerful components of both global and national responses to the COVID-19 pandemic.

## 1 Introduction

The whole world has been closely focusing on an outbreak of respiratory disease caused by a novel coronavirus first reported in Wuhan, China, on December 31, 2019, as it continues to spread at an alarming rate. On February 11, 2020, the World Health Organization (WHO) named the disease “coronavirus disease 2019” (COVID-19). COVID-19 poses significant threats to international health. Similar to the flu, COVID-19 is thought to spread mainly between people who are in close contact with one another through respiratory droplets produced when an infected person coughs or sneezes. After COVID-19 was confirmed to be spread by human-to-human transmission on January 20, 2020, the government immediately launched the first-level response to major public health emergencies. On January 30, 2020, the WHO declared this ongoing outbreak as a global public health emergency and raised the global risk of COVID-19 to very high on February 28, 2020.^[1]^

CT can play an important role in both diagnosing and follow up of COVID-19 based on case definitions issued by the WHO and the treatment guidelines from the National Health Commission (NHC).^[2]^ Patients who are suspected or confirmed to have the virus may undergo chest CT. Due to overlaps in the imaging findings with other respiratory diseases, CT is not helpful as a screening tool. However, it can help identify the degree of pulmonary involvement and disease course.

“Wuhan Living room" makeshift hospital, a kind of emergency hospital shelter employed to combat coronavirus, is called a “Fangcang” shelter hospital. They were first implemented in China in February 2020 and served to isolate patients with mild to moderate COVID-19 from their families and communities, while providing medical care, disease monitoring, food, shelter, and social activities. In February 2020, the NHC and relevant units established Wuhan Fire God Mountain Hospital, Wuhan Thunder God Mountain Hospital, and 13 fangcang shelter hospitals in Wuhan. Wuhan international convention and exhibition center, as well as gymnasiums and schools, have been selected as the best places for a makeshift hospital. According to the situation of epidemic prevention and control, all emergency shelter hospitals had been closed until March 10, 2020.

This article describes advice and recommendation on a radiology management procedure and the infection-protection experience of the department staff in makeshift hospitals in Wuhan.

## 2 Materials and methods

### 2.1 Confirmed mild to moderate patient

Our institutional review board approved this single-center retrospective study with a waiver of informed consent, and the study was approved by the Medical Ethics Committee of the First Hospital of Jilin University (Changchun, China; Approval No. AF-IRB-026-01). Fangcang shelter hospital admission criteria are as follows^[3]^:

(1) confirmed patients identified according to the Diagnosis and Treatment Program of the Novel Coronavirus Pneumonia of the NHC. The real-time reverse transcription polymerase chain reaction test used to detect SARS-CoV-2 nucleic acid is positive, and viral gene sequencing analysis is highly homologous with SARS-CoV-2.
(2) Mild and moderate cases only show mild signs or symptoms, such as low-grade fever, mild fatigue, dry cough, and no signs of pneumonia, and a few patients have symptoms such as nasal congestion, runny nose, sore throat, myalgia, and diarrhea.
(3) All patients in the makeshift hospital can walk and live independently.
(4) Absence of severe chronic diseases, including hypertension, diabetes, coronary heart disease, malignancy, structural lung disease, pulmonary heart disease, immunosuppression, and mental disease.
(5) Blood oxygen saturation > 93% and breathing rate < 30 beats per min in a resting state.
(6) Age range 18 to 65 years old.

### 2.2 Human resources allocation and personal protection

Nurses have more experienced and professional knowledge in protection and infection control, while technologist are more experienced at scanning and image processing. In a temporary adiology department in a makeshift hospital, there are scanning posts (technician), auxiliary scanning posts (technician), and guide posts (nurses). The scanning post is responsible for patient scanning, image observation, and communicating with the diagnostic doctor. The auxiliary scanning post is mainly responsible for checking information and patient positioning. This guide post is mainly responsible for reception, inquiry, and infection control. The whole process was decreased from 8 minute to 4 minute, which greatly reduced the waiting time for patients and the detention time in the radiology department and improved the circulation rate of patients. Because staff members working in the contaminated area are under much situational pressure, periodically taking time off could lower their physical and mental stress levels. The technologists on fever-CT duty shifts are provided a break once every two days for 4 to 6 hours. In addition, the health of staff in the contaminated area must be monitored closely for the symptoms of COVID-19.

Health care workers are at high risk of infection of COVID-19, so all hospital staff who work in makeshift hospitals are required to wear complete personal protective equipment^[4]^: medical protective clothing, surgical cap, N95 mask, gloves, face shields, and goggles. Wearing and removing the equipment must be performed following proper procedures and under the supervision of the infection control nurse.

### 2.3 Configuration of the radiology region

**Figure 1:**
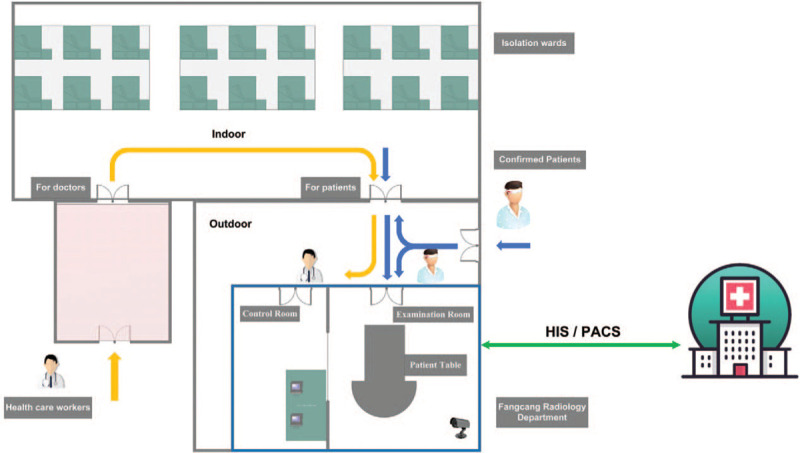
Schematic overview of Hangjiang Fangcang shelter hospital (indoor, isolation wards) and shelter radiology department (outdoor). Contaminated (shaded in white), semicontaminated (shaded in light pink).

The radiology region should be configured, taking into consideration the following: (1) Reduce the risk of infection among health care workers. The whole area is divided into the contaminated area, semi-contaminated area, and clean area (Fig. 1). The contaminated area contains the examination room, the CT operation room, and the access to the operation room. The cleaning area is the activity area of the staff, such as the diagnostic room. Diagram of the layout of the temporary makeshift radiology department in Wuhan Hanjiang shelter hospital was shown in Figure 1. (2) Uncontrollable factors, such as the weather, and the surrounding environment should be considered. We recommend that the radiology region should not be located far from the patient channel, but should be as far as possible from the doctor channel. It is best to choose an outdoor venue instead of an indoor one. At the same time, anti-cold anti-skid measures must be prepared in advance. (3) Considering site and equipment factors, the Fangcang shelter hospitals in China were large-scale, temporary hospitals, rapidly built by converting existing public venues, such as stadiums, convention centers, exhibition centers, gymnasiums, schools. Different sites have different designs. Besides location, equipment factors should be considered. There are two common device types for CT examination in this example. One is called “Fangcang CT,” which is fixed in a specific area with radiological protection and is large enough to build separate accesses for doctors and patients to avoid cross-infection. The other option is a mobile vehicle CT. This kind of equipment has space-saving advantages and convenient transportation, but it is not open air. Furthermore, for people with mobility problems, getting into a mobile vehicle CT examination table is challenging.

### 2.4 Radiology examination procedures

**Figure 2:**
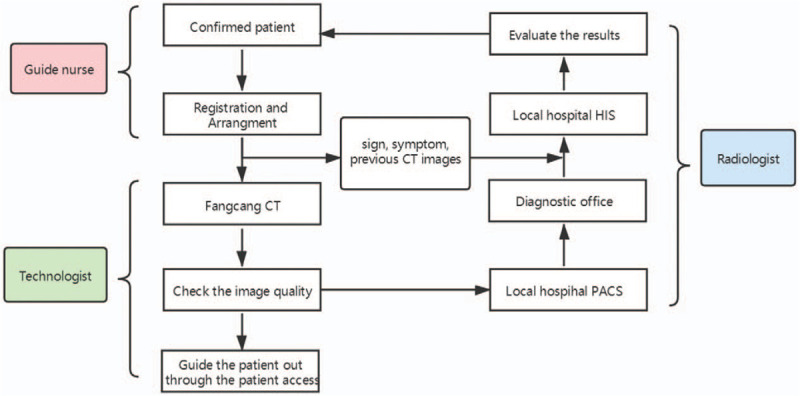
Fangcang CT examination protocol for confirmed patient with COVID-2019. COVID-19 = coronavirus disease 2019, CT = computed tomography.

When the patient makes an appointment for examination, the staff asks the patient about their epidemiological history, symptoms, signs, and collects the results of any previous CTs before the examination. The patient uses the patient access area to enter the CT waiting area. If the patient can get onto and off the examination table by themselves, they are allowed to do so. If the patient cannot independently get onto or off the examination table, the person accompanying the guide nurse, not the technologist, assists the patient. The auxiliary technologist checks the patient information and uses an intercom system in the examination room to ask the patient to remove any metal ornaments on the neck and chest. Furthermore, the technologist trains the patient to hold their breath during the examination. After scanning, the original images are reconstructed as 1 mm-thick layers. The technologist browses the images to ensure that their quality meets diagnostic requirements and then informs the patient to leave through the patient access area. The radiologist staying in a clean diagnosis office will make the diagnosis and compare CT imaging results to previous records. The radiologist on duty will notify clinicians upon discovery of radiological abnormalities or patterns that portend a poor outcome or obvious progression. One COVID-19-infected patient with the highest severity score (lung involvement score) on the CT scan was admitted to the intensive care unit.^[5]^ The equipment was disinfected according to the procedure described under Equipment and Environment Disinfection Procedures. Figure 2 shows the CT examination protocol for a confirmed patient with COVID-19.

### 2.5 Equipment and environment disinfection procedures

The Routine Disinfection Procedure included

(1) object surface disinfection: the object surface is wiped with 1,000 mg/L chlorine-containing disinfectant, wiped twice with 75% ethanol to avoid corrosion, once every 4 hours.
(2) Equipment disinfection: The equipment in the contaminated area is wiped with 2,000 mg/L chlorine-containing disinfectant. The DR and CT gantry in the contamination area is wiped with 75% ethanol. The examination bed and ground are disinfected with 2,000 mg/L chlorinated disinfectant once a day.
(3) Air disinfection: The air conditioners were turned off to prevent air contamination through circulation. In contaminated areas, the door is opened once every 4 hours to ventilate for over 30 minute.

## 3 Results and discussion

The Hanjiang shelter hospital and Hanyang Sports School shelter hospital came into service in Wuhan, China, from February 11, 2020 to March 10, 2020. We counted the number of patients admitted to the two hospitals and radiological related statistics (shown in Table 1). The maximum number of daily CT examinations reached 289. The CT scanned a patient approximately every 13 minute. As a result of our precautions, none of the staff of the radiology department developed symptoms suspicious for COVID-19. The Fangcang CT technologist, with the highest probability of exposure, remains negative per polymerase chain reaction.

**Table 1:** Fangcang shelter hospital characteristics.

	Hanjiang Fangcang shelter hospital	Hanyang Sports School Fangcang shelter hospital
Maximum admission	2000 patients	960 patients
Total number of patients admitted	1848 patients	300 patients
Total number of CT examination	2730	510
Device type	2 CTs and 1 DR	2 CTs
Number of radiologist/technologist	4/16	1/11

CT = computed tomography, DR = Digital Radiography.

Compared with the speed and extent of the severe acute respiratory syndrome, the spread of COVID-19 all over the world over four months is unprecedented. This spread has been facilitated by its contagious nature and rapid spread via droplets and person-to-person contact. The droplet mode of transmission means that a person can be infected easily through casual contact or even fomites on contaminated environmental surfaces.

The experience with Fangcang shelter hospitals during the COVID-19 outbreak in China suggests that they could be powerfully employed in future public health emergencies as well as other events involving illness or injury on a large or rapidly growing scale. The Fangcang shelter hospitals mainly consist of a medical cabin, technical support cabin, ward unit, life support unit, and transportation cabin. Furthermore, they can provide large numbers of hospital beds and appropriate care for patients who do not have severe or critical diseases. In order to reduce the number of asymptomatic or mild patients causing disease spread, and to save limited medical resources, this concept of the makeshift hospital was introduced into resisting COVID-19. The makeshift radiology department is the core of the hospital, and mobile CT plays an irreplaceable role in patient management and efficacy evaluation. However, The COVID-19 guideline of the NHC does not recommend chest DR, because its ability to diagnose COVID-19 is limited. Placing the radiation region in the open outdoor area is a good choice to facilitate the passage of patients and reduce cross-infection. We recommend that the radiation region be equipped with an examination room and an operation room, which will have dedicated separate accesses for doctors and patients.

How radiology departments respond to any infectious disease outbreak should consider the estimated risk of the disease, and radiologists should change methods according to practical clinical work and the variable situation. All typical and atypical imaging features of the disease should be made known to all radiologists to assist in recognition of the disease on images and to allow accurate reporting of these findings. Collaboration with the radiology departments of other hospitals is crucial. Therefore, we suggest that the Picture Archiving and Communication Systems and Hospital Information System of the makeshift radiology department be supported by cloud platforms and connected with higher-level local hospitals. This makes record-keeping and image storage convenient for all probable cases of COVID-19 makes available images from previous imaging studies for comparison. In makeshift hospitals, the staff is in direct contact with patients. Therefore, the strongest precautions need to be implemented to limit the cross-infection to the staff and confirmed patients. In addition to contact minimization for chest CT and DR examinations, the timely disinfection of CT and DR examination rooms should be appropriately implemented.

The Internet and social media apps, especially WeChat, have been used for distributing medical information, sharing up-to-date guidelines, and communicating with patients or colleges. It is an indispensable tool for radiologists to present the CT manifestations of COVID-19, which was completed in other hospitals. We suggest implementing individual cellular phones for work and not using personal ones on duty.

In the makeshift hospitals mentioned in our study, we adopted the low dose chest CT scan protocol. The low dose CT plays an important role in understanding the extent and dynamic evolution of lung lesions induced by COVID-19. Many CT examinations may be conducted in the same individual to monitor disease progress, and low-dose scanning can reduce the radiation damage to patients. With the advantages of an improved detector, high pitch settings, lower tube voltage (80–100 kVp) and current (10–25 mAs), iterative reconstruction, and dose reduction options, it is now feasible to minimize the radiation dose. Kang et al.^[6]^ recommended low-dose CT in the detection and management of COVID-2019; they implemented a low-dose scanning protocol that reduced the patient's dose to 1/8 to 1/9 of the standard CT dose.

This study had some limitations. The number of study subjects was limited. Ideally, statistical datasets should be implemented in all makeshift radiology departments in China. However, we choose two typical models and describe many aspects in detail, including site distribution, human resources, and workflow. We hope that this valuable experience will help radiologists around the world who are facing the same problems.

In conclusion, strategic planning and adequate protections can help protect patients and staff against a highly infectious disease. Aspects of the Fangcang shelter radiology department could be powerful components of national responses to the COVID-19 pandemic, as well as future public health emergencies.

## Author contributions

**Conceptualization:** Yun Pei, Jingyu Wang.

**Data curation:** Lin Mu, Jingyu Wang.

**Formal analysis:** Yun Pei, Jingyu Wang.

**Investigation:** Yun Pei.

**Methodology:** Caijuan Zhang.

**Project administration:** Caijuan Zhang, Yun Pei.

**Resources:** Yun Pei.

**Software:** Lin Mu, Caijuan Zhang.

**Supervision:** Lin Mu, Jingyu Wang.

**Validation:** Lin Mu, Caijuan Zhang, Jingyu Wang.

**Visualization:** Jingyu Wang.

**Writing – original draft:** Lin Mu, Caijuan Zhang, Jingyu Wang.

**Writing – review & editing:** Jingyu Wang.
